# PAPAyA: a platform for breast cancer biomarker signature discovery, evaluation and assessment

**DOI:** 10.1186/1471-2105-10-S9-S7

**Published:** 2009-09-17

**Authors:** Angel Janevski, Sitharthan Kamalakaran, Nilanjana Banerjee, Vinay Varadan, Nevenka Dimitrova

**Affiliations:** 1 Philips Research North America, 345 Scarborough Road, Briarcliff Manor, NY10510, USA

## Abstract

**Background:**

The decision environment for cancer care is becoming increasingly complex due to the discovery and development of novel genomic tests that offer information regarding therapy response, prognosis and monitoring, in addition to traditional histopathology. There is, therefore, a need for translational clinical tools based on molecular bioinformatics, particularly in current cancer care, that can acquire, analyze the data, and interpret and present information from multiple diagnostic modalities to help the clinician make effective decisions.

**Results:**

We present a platform for molecular signature discovery and clinical decision support that relies on genomic and epigenomic measurement modalities as well as clinical parameters such as histopathological results and survival information. Our **P**hysician **A**ccessible **P**reclinical **A**nal**y**tics **A**pplication (PAPAyA) integrates a powerful set of statistical and machine learning tools that leverage the connections among the different modalities. It is easily extendable and reconfigurable to support integration of existing research methods and tools into powerful data analysis and interpretation pipelines. A current configuration of PAPAyA with examples of its performance on breast cancer molecular profiles is used to present the platform in action.

**Conclusion:**

PAPAyA enables analysis of data from (pre)clinical studies, formulation of new clinical hypotheses, and facilitates clinical decision support by abstracting molecular profiles for clinicians.

## Background

### Introduction

Advancement in molecular bioinformatics research is generating an overwhelming amount of information. Clinicians acknowledge that there is a need to accelerate the translation of knowledge discovery from genome scale studies to effective treatment and tailored cancer management. Commercially available tools such as GeneSpring or open source tools can process and visualize genomics data for preclinical applications. On the clinical side, a number of clinical decision support tools exist that incorporate clinical guidelines, assist clinicians in diagnostics, or intelligently interpret clinical data to give insight in the underlying trends. However, there is unique clinical value to be added by providing the clinician with an integrated view of the patient molecular profile and where the patient is compared to patients with similar clinical parameters and history. For the latest genomic tests that have entered the clinical guidelines, there is need for clinician driven analysis with patient-centric data and informatics-assisted discovery in an easily configurable environment that could be quickly tuned to new clinical questions.

There is a dearth of tools focused on the clinical use scenario that can meaningfully integrate information from multiple molecular modalities such as genomic (copy number variation), transcriptomic (gene expression) and epigenetic (differential methylation) data and that can provide a clinically-relevant comprehensive picture of the molecular state of a sample. Systems exist that acknowledge this issue and integrate various molecular data [1], however such work is primarily driven by knowledge discovery rather than for clinical use.

In this paper we introduce a Physician Accessible Preclinical Analytics Application – PAPAyA, a platform for clinical decision making that relies on multiple information modalities: gene expression and differential DNA methylation as well as clinical parameters such as histopathological results and survival information. We have assembled a powerful set of statistical and machine learning tools that leverage the connections among the different modalities and present a clinically meaningful portrait of the individual sample.

### Breast cancer and genomic profiling

Breast cancer is a complex disease driven by the accumulation of multiple molecular alterations. Recent molecular advances in high-throughput genomic, transcriptomic and epigenomic technologies have made it possible to focus on the molecular complexity of breast cancer and help guide cancer prognostication and therapy prediction.

Perou et al. demonstrated that breast cancer can be classified into distinct groups based on their gene expression profiles [2]. The Estrogen Receptor positive (ER+) group is characterized by higher expression of a panel of genes that are typically expressed by breast luminal epithelial cells ('luminal' cancer). The Estrogen Receptor negative (ER-) branch covered three subgroups of tumors: 1) overexpressing ERBB2 (HER2); 2) expressing genes characteristic of breast basal cells (basal-like cancer); and 3) normal-like samples. The clinical relevance of this stratification is that ER+ tumors are typically associated with good prognosis and basal-like and HER2 tumors have poor prognosis. Further refinement in molecular classification however, can result in differing clinical significance.

Gene expression profiling has also led to the development of several gene-expression assays, of which Onco*type*DX [3] and MammaPrint [4] are gaining acceptance in routine clinical use. Onco*type*DX analyzes the expression of 21 genes and calculates a recurrence score to identify the likelihood of cancer recurrence in patients and an assessment of their likely benefit from chemotherapy. MammaPrint analyzes the expression of 70 genes and allows patients (<61 years) with early-stage breast cancer to be categorized as having risk of distant metastasis. High-risk patients may then be managed with more aggressive therapy, while low risk patients can be spared from toxic chemotherapy.

Recent advances in molecular profiling technologies have led to the application of more than one genomic modality to address similar clinical questions. For example, gene expression profiling can be enhanced with the detection of certain genomic copy number variations, amplifications and deletions using Representational Oligonucleotide Microarray Analysis (ROMA) and correlated with patient survival [5]. We have also co developed Methylation Oligonucleotide Microarray Analysis (MOMA) in collaboration with Cold Spring Harbor Laboratory to perform genome-wide scans of CpG island methylation in normal and tumor samples [6].

Such growth in genomic profiling strategies leading to additional in-vitro diagnostic multivariate index assays will result in a plethora of tailored genomic tests that cater to specific clinically prevalent sub-populations. Appropriately integrating the information provided by multiple genomic profiling strategies can help reduce the resulting complexity in decision-making and offer unique patient-specific insights to the clinician. The main objective in designing the PAPAyA platform was to provide a highly flexible translational platform where we can explore integration of patient clinical data and multiple high-throughput molecular measurements. The aims were to design and implement a platform that can a) easily be used to prototype new ideas for clinical studies support, and b) introduce new clinical tools for data analysis contexts relevant to clinical practice based on molecular measurements.

## Methods

### Design

PAPAyA is designed to provide common interface to multiple data sources, use of tools that utilize the data, and to facilitate a flow of data analysis and assessments of results. PAPAyA stores and provides access both to clinical as well as molecular data from clinical studies. The user interface enables access and analysis of data from multiple samples in a discovery flow, and clinical interpretation flow on a single sample – a patient. The core data access and presentation functionality is built in the platform, whereas the available data transformation steps are dynamically defined through plug-in tools depending on the data type and use flow.

The design supports easy registration and execution of applications written in R, Matlab, Python, and Perl as well as binary executables. It is in principle easy to extend this support to additional execution platforms. Applications are registered as tools annotated for their use contexts, which enable definition of numerous analysis pipelines capturing a sequence of processing steps. Tools can be developed for a specific modality (e.g. copy number variation, gene expression, and methylation) or for a particular clinical study. Here, *context *is a collection of tools that are allowed to be invoked at a certain step in the workflow.

### Principal architecture

**Figure 1:**
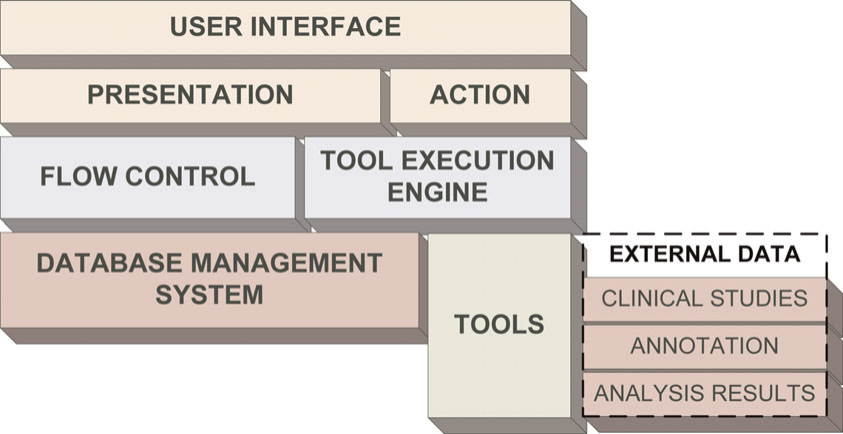
**Principal architecture of PAPAyA**. *User Interface *combines elements of presentation and handles user actions. *Presentation *shows synchronously the present context(s), data, analysis results, output of tool executions, and visual elements for navigation and execution dynamically based on the current application context. *Flow Control *translates the context definitions into visualization components. Based on user's interaction, *Action *translates user requests into a change of display (and context) or requests a tool execution by the *Tool Execution Engine*. The latter component, based on the application context, user-provided input and the description of the tool handles the output. The execution instance of a *Tool *is controlled by the application to the extent that it provides parameters for its execution. Tools have access to the internal database, but given the loose coupling with PAPAyA, it is often the case that *External Data *is used by a tool. Such data comprise measurements, annotation, and results from the execution of other tools within PAPAyA.

The principal architecture of the platform is given in Figure 1. *User Interface *combines elements of presentation and handles user actions. *Presentation *shows synchronously the present context(s), data, analysis results, output of tool executions, and visual elements for navigation and execution dynamically based on the current application context. *Flow Control *translates the context definitions into visualization components. Based on user's interaction, *Action *translates user requests into a change of display (and context) or requests a tool execution by the *Tool Execution Engine*. The latter component, based on the application context, user-provided input and the description of the tool handles the output. The execution instance of a *Tool *is controlled by the application to the extent that it provides parameters for its execution.

The salient feature of PAPAyA is that not only data from the studies but also application behavior and tool definitions are stored and handled by the comprehensive *Database Management System*. Given the intentional loose coupling of each tool with the platform, it is typical to assume that each tool comes with its own data – *External Data*. However, many of the tools utilize the data stored in PAPAyA's database.

### PAPAyA building blocks

#### Contexts

The behavior of PAPAyA is defined by the user through a state diagram where each state can have multiple contexts. Transition from one context to another is defined a priori by some user or tool action. PAPAyA allows for fine-tuning of context descriptions with user-defined constraint variables, which are set and un-set depending on the user's actions and selected element type as in sample ID, measurement modality, or microarray probe. The visual representation of the contexts consists of two components: a display of the relevant parameters of the selected sample, measurements, measurement feature, signature, etc., and dynamic access to the available tools in the current context.

#### Execution

Each tool is an application or a routine that is defined by its execution platform (e.g. R, Matlab), output type (e.g. graphics or text), and parameters that the GUI handles by providing the user with a dialog to fill in. The input parameters can be pre-filled from the tool definition, but also from the current context of execution (e.g. the current patient ID, sample ID). It is very easy to integrate a tool into PAPAyA. Most software modules will likely require no modification apart from formatting the output to comply with the visual elements of the user interface. This also enables improved versions of the tools to be added by simply replacing the current tool with an updated version without reconfiguration of PAPAyA.

#### Data

An instance of PAPAyA needs to be configured to support the data requirements and the user need for transformation of the data. This is done through flow definition in conjunction with registration of tools. The data structure in Table 1 defines flows by characterizing states and contexts that are linked to the tools' definitions. Each row in the *flow table *defines a *transition *which encodes different states of user interaction and their changes. Several states are pre-defined and are internal to the platform – they link to the static part of the user interface. The user is allowed to configure any number of user-specified states characterized by some transition. A transition is typically an invocation of a tool described in a row of the *flow *table. In addition, transition can set or reset internal constraint variables that together with the current state, define the current *context*. In each state, the context determines which transitions are available to the user at a particular step (time). Based on this, the user interface dynamically configures the layout of the screen as well as the visual elements that allow the user to activate the permitted transitions.

**Table 1:** Flow Definition.

***Field***	***Description***
StateFrom	Current state
Type	Tool execution; internal transition; ...
Description	Free-text description
Action	Tool name; initialization; internal actions
ConstraintExist	User-defined variables that can be set to define constraints. For example methylation modality active vs. expression modality active; analysis mode vs. decision support mode; etc.
ConstraintSet	Constraints to set with this transition
Constraint-Unset	Constraints to unset with this transition
StateTo	New state

The flow is defined with transitions between states (from StateFrom to StateTo) based on use Action. Example state may be pre-processing, analysis, post-processing, and single-patient evaluation. Each state uses internal constraints that can be set or unset during transitions (based on ConstraintSet and ConstraintUnset). The current state and the constraints currently set (ConstraintExist) define a context. Example constraints can be based on the molecular modality used. For example, constraint EXP (for expression) and AFFY (for an Affymetrix platform) when set in the pre-processing state will make available only the tools suitable for the platform and modality.

#### Implementation

PAPAyA is implemented in C#. The GUI-intensive parts of PAPAyA enable navigation through clinical studies where the central part is the patient sample. For each sample, all available measurements are navigable and linked to further characterizations available in the context of the sample and the modality. In PAPAyA, all characterization is around molecular signatures as described in more detail in the Results section. Furthermore, PAPAyA provides decision support views of the current patient sample and measurements that transform the results for use in a clinical setting.

## Results

### Overview

The focus of PAPAyA is on discovery of molecular signatures for clinical stratification of patient samples and their utilization in a clinical setting. The data browsing and signature discovery pipeline comprises statistical and machine learning algorithms (supervised and unsupervised) and operates on multiple modalities of high throughput measurements in a high-performance computing environment. The output of these algorithms consists of molecular signatures addressing specific clinical questions (benign vs. malignant, tumor subtype, relapse free survival, etc.) that are based on several individual or combination of modalities (DNA copy number, DNA methylation, and gene expression). The signatures are typically evaluated for performance characteristics (sensitivity and specificity) using statistical approaches. Clinical researchers can benefit from an integrated system that enables them to evaluate and explore these signatures in a user-friendly environment, and be able to characterize signatures and the likely scenarios in which they can be integrated into an oncologist's or pathologist's clinical practice.

PAPAyA facilitates these tasks by applying multivariate statistical analysis and data mining algorithms across modalities in an integrated fashion. The clinical trial database is accessible to bioinformatics tools such as feature filtering, hierarchical clustering, multimodal feature correlation, top-down hierarchical sorting, a methyl binding sites tool, and computationally intensive search approaches such as our CHC Genetic Algorithm (GA) coupled with Support Vector Machines [7]. These tools are used for discovery of univariate and multivariate prognostic or predictive signatures and clinically relevant disease subtypes using each of the modalities independently and in combination.

PAPAyA allows for patient-centric analysis and informatics-assisted discovery to be performed systematically in a pipeline that is fine-tuned to assist in answering specific clinical questions. The analysis can be tailored to be patient-centric or signature centric thereby allowing for discovery or clinical decision support respectively. As a way of introduction to our platform we present several use scenarios of integrated analysis of multi-modality retrospective breast cancer data [2,5,8].

### Discovery using PAPAyA

**Figure 2:**
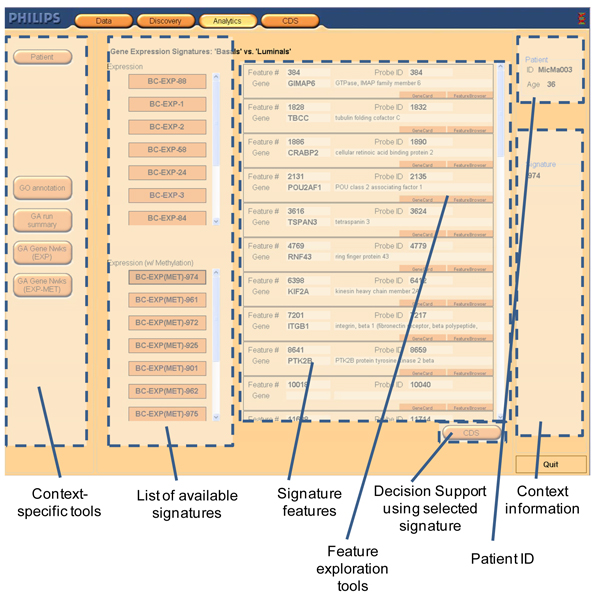
**Summary and exploration of signatures**. Different tools and functionalities are highlighted in the dotted boxes. Output from tool execution is shown in the central part of the screen. Context-specific tools and Context information remain on the screen at all times.

In discovery, high-throughput molecular data is processed using a series of tools that are available to the user as the analysis progresses. Initially, the user selects one of the filtering tools and based for example on selection of gene expression data, the context of the analysis will prompt PAPAyA to make available a set of tools such as a genetic algorithm wrapper around a classifier designed for gene expression data. This wrapper tool can be executed multiple times using different configurations using a different type of classifier to evaluate candidate signatures. The result of this analysis is a set of candidate signatures that the researcher can analyze and prioritize based on the application of additional available tools in this context. Figure 2 shows an annotated screenshot of the PAPAyA interface to a list of signatures that classify the patient as belonging to either luminal or basal subtype. Here, one of the candidate signatures is expanded to obtain information on their member genes with access to gene cards and feature browser.

**Figure 3:**
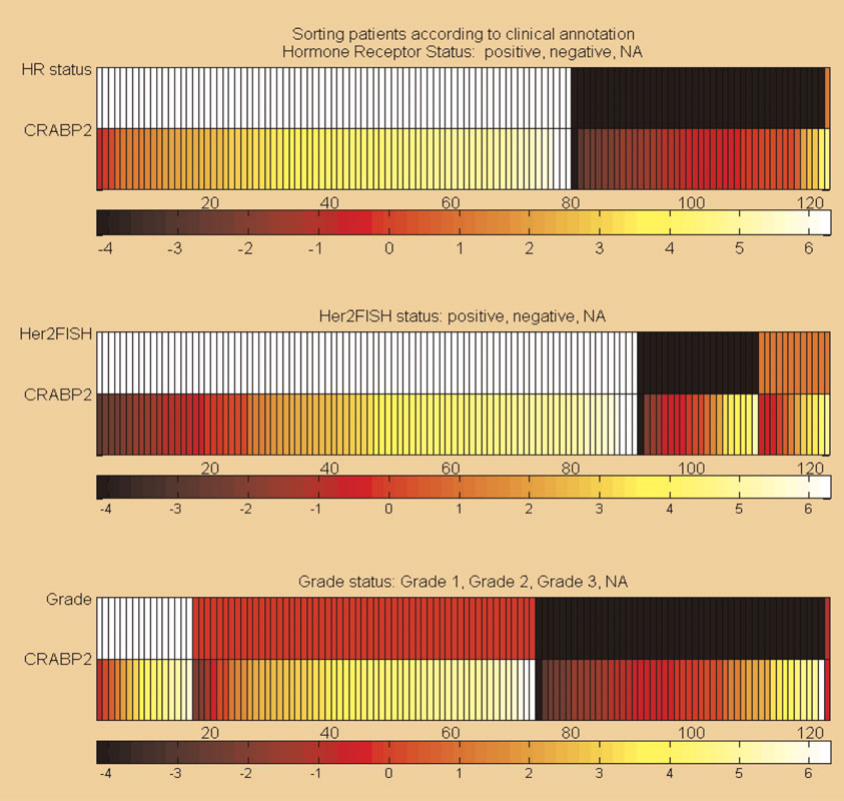
**The Feature Browser tool helps visualize correlations between gene expression levels and clinical parameters**. Example of clinical data shown with the expression of CRABP2 (cellular retinoic acid binding proteins – a carrier protein for members of the vitamin A family). Three heatmaps show the expression for this gene grouped based on the clinical information. In the first heatmap, hormone receptor status is used to group the samples under positive (white bars in the first row) and negative (black bars in the first row). In the second heatmap, HER2 status is used to group the HER2 positive samples (white bars), and HER2 negative (black bars). The third heatmap groups the samples based on grade: grade I (white bars), grade II (orange bars), and grade III (black bars). This gene's expression level correlates with the hormone status (under expressed in hormone positive and over expressed in hormone positive) and tumor grade (over expressed in grade I and II, and under expressed in grade III). This tool thus enables the user to gain insights into clinical associations that were not used in the signature discovery process. The tool also can also generate heatmaps for any number of genes, allowing joint assessment of the expression for multiple expression profiles.

Discovered gene expression signatures can now be further explored using the signature browser as a starting point. The signature browser allows the user to navigate among the various signatures, their component genes and performance. Most importantly, based on the current context, PAPAyA makes the appropriate tools available to the user in a seamless manner. For example, a tool to organize gene expression heatmaps by histopathological parameters such as grade, ER status is one such tool. This visualization provides an additional level of detail in browsing specific gene expression levels. Figure 3 shows the output of this tool. The user will observe that the gene's expression level also correlates with the hormone status (under expressed in hormone positive and over expressed in hormone positive) and tumor grade (over expressed in grade I and II, and under expressed in grade III). This tool thus enables the user to gain insights into clinical associations that were not used in the signature discovery process. This helps to assess significance of the genes as well as confidence in the signatures in which these genes are found.

**Figure 4:**
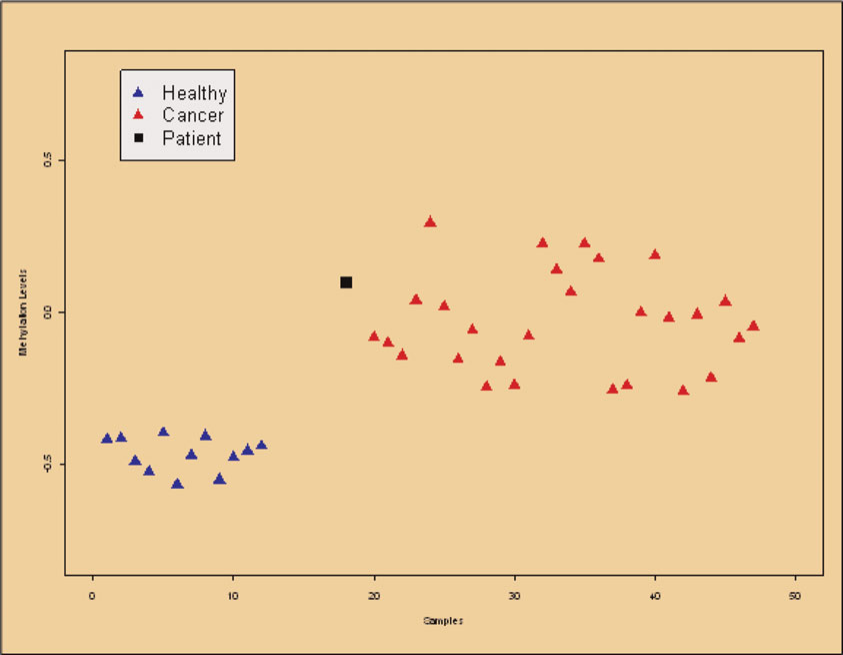
**Methylation feature browser**. The DNA methylation of a single locus is a diagnostic marker from breast cancer. The level of DNA methylation is indicated on the y axis, whereas the x axis enumerates the samples. Here, all normal samples are not methylated, and the cancer samples are. The point marked with a black square indicates the methylation level of a sample of interest – this is used to assess a methylation profile in the context of a clinical study.

The signature browser also links to tools that extend the analysis to additional modalities for which data is available. For example, when DNA methylation data is available for the same sample set, the methylation profiles of individual genes can be directly accessed and visualized. Starting with methylation based signatures in the signature browser, the user can explore the underlying DNA methylation profiles of the loci that constitute methylation signatures. Similar to the gene expression example in Figure 3, DNA methylation data can be organized around clinical groups, thus providing additional information to the user. Figure 4 shows the methylation state of one such locus in a set of breast cancer samples stratified into normal and tumor tissues. This tool can also be used in a clinical setting to assess the confidence in patient stratification. In Figure 4, the specific patient profile is shown together with the samples from a related clinical study and the measurement for this patient for this locus is marked. Here, there is indication that the methylation profile of this patient may be more similar to the normal samples rather than tumors into which this patient is placed according to the diagnosis. Such indication and other stratifications can be further used by the clinician to drive for example therapy decisions.

Thus PAPAyA user interface provides the user with the applicable tools based on the selected modality and stage in the discovery process. Another important aspect of this process is that the entire framework driving it is easily extensible with additional tools and data, maintaining the capability to work across multiple modalities. Therefore, additional data collected for the same samples, (Eg. microRNAs profiling or sequencing/mutation information) can be easily added to the analysis by adding tools and contexts to the flow to support visualization and analysis of such data.

### Clinical decision support

**Figure 5:**
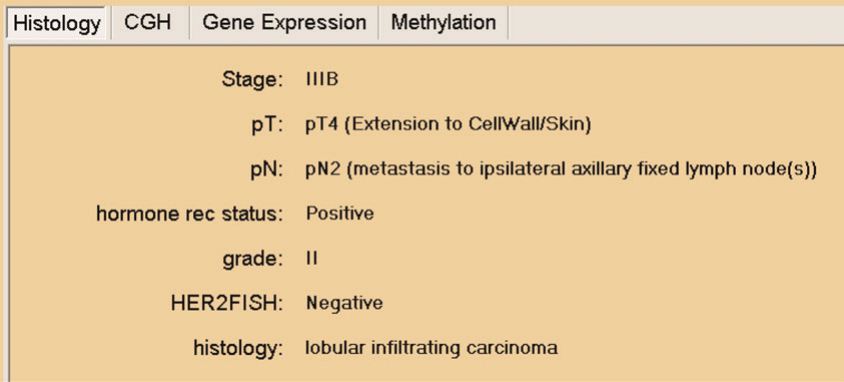
**The Clinical Data Explorer**. Access to clinical data for a patient such as stage, grade ER and ErbB2 status.

**Figure 6:**
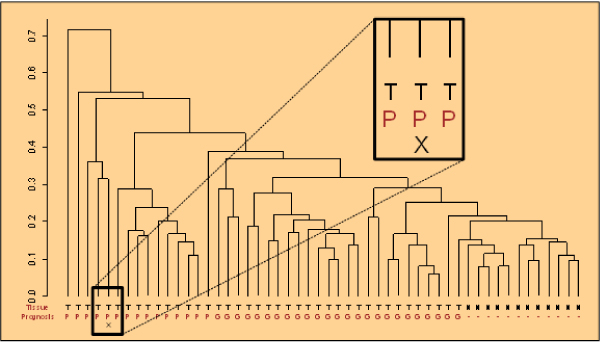
**Clinical decision support from DNA methylation signatures**. One patient's profile is visualized against all samples in a clinical study in the form of a dendrogram. The leaves of the dendrogram are annotated with tumor/normal, and good/poor prognosis. In the figure inset, the patient is marked with an 'X'.

**Figure 7:**
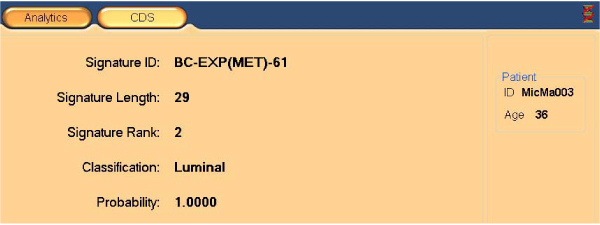
**Clinical decision support from gene expression signatures**. The gene expression signature that best predict the cancer subtype for a patient is shown with its prediction probability.

The same concepts as presented above are used in the clinical decision support (CDS) mode of PAPAyA assists in the interpretation of tumor profiles of specific patients. In a patient-centric mode, we start by browsing the basic clinical data such as tumor size, stage and grade, and histopathological data such as hormone receptor status (estrogen receptor ER and progesterone receptor PR) and ErbB2 amplification as shown in Figure 5. The clinical data explorer provides access the molecular profiling data as well as the signatures derived from high-throughput modalities such as gene expression, DNA methylation and copy number measurements. Signatures derived in the discovery process are applied to stratify the patient samples and can be used to assign confidence in a stratification based on the signature's performance with respect to all patients in the database. We present output from PAPAyA platform's CDS modules based on Support Vector Machine based classifiers for predicting tumor subtype using gene expression and clustering of DNA methylation profiling data. In Figure 6 shows the results of a hierarchical clustering algorithm used to stratify samples based on their DNA methylation profiles. The patient's profile is visualized against all samples in a clinical study in the form of a dendrogram and the figure inset shows the patient sample marked with an 'X'. The homogeneity of the subtree in which the patient's sample appears provides the clinician an indication of the confidence of the prediction. In another example, to facilitate clinical decisions based on the gene expression signatures, an (support vector machine) SVM classifier provides prediction of cancer subtype along with a measure of confidence in the prediction. In Figure 7, we provide part of a screenshot showing the output of this CDS tool, where the subtype prediction for the patient's sample along with the prediction probability is indicated.

The statistics and visualization we just described can be used by clinicians to gain additional insights and tailor the treatment to the physiological state of the patient. Tools that implement additional standard tools such as breast cancer clinical prognostic indices such as Nottingham Prognostic Index and St. Gallen Consensus can also be easily incorporated into PAPAyA. Additionally, integration of third party molecular signatures into the platform is supported by the existing database structure.

## Discussion

We designed and implemented PAPAyA as a platform that can easily be used to integrate existing tools and facilitate prototyping new ideas for clinical studies support. It also provides new clinical tools around multiple molecular modalities, standard clinical parameters and contexts defined by clinical experts. The platform has flexible architecture and can incorporate new modalities very easily. This flexibility still introduces different practical challenges. For example, quality control of new tools or tool updates is essential as well as ensuring appropriate combinations of tools to avoid derivation of wrong conclusions (e.g. use protocol definitions).

To leverage the insights into the molecular state of clinical samples, there has to be clinically-relevant linkage across modalities. We have started deep integration of DNA methylation and gene expression in PAPAyA, however we have to further include tools that facilitate integration of the inherent dependencies between the molecular and the standard histopathological modality. Finally, it would be extremely useful to integrate imaging data and utilize tumor morphology and texture with the molecular signatures for applications such as prognosis and prediction.

## Competing interests

The authors declare that they have no competing interests.

## Authors' contributions

All authors developed the concepts, design, and specification jointly. All authors wrote the article. All authors implemented tools integrated in the platform. AJ implemented the software and the database.
